# THE PROMINENT ROLE OF HOME CARE AIDES IN END-OF-LIFE CARE DURING A PUBLIC HEALTH EMERGENCY AND BEYOND

**DOI:** 10.1093/geroni/igad104.0627

**Published:** 2023-12-21

**Authors:** Patricia Kim, Emily Franzosa, Laura Moreines, Jonelle Boafo, Daniel David, Abraham Brody, Melissa Aldridge

**Affiliations:** Icahn School of Medicine at Mount Sinai, New York City, New York, United States; Icahn School of Medicine at Mount Sinai, New York City, New York, United States; New York University, New York City, New York, United States; Rory Meyers College of Nursing, New York City, New York, United States; NYU Rory Meyers College of Nursing, New York City, New York, United States; NYU Rory Meyers College of Nursing, New York City, New York, United States; Icahn School of Medicine at Mount Sinai, New York City, New York, United States

## Abstract

Many patients and families rely on paid aides to provide personal care services at the end of life, but the COVID-19 pandemic caused widespread delays and disruption in this care. Our study examined COVID-related care disruptions among patients in a large, New York City health system who died between March 1, 2020 and March 1, 2021. We linked the electronic health records of 124 patients cared for by a geriatric outpatient practice and referred to hospice to the hospice’s electronic health records, and conducted an in-depth qualitative thematic analysis. We found many home health aides continued caring for existing patients after hospice enrollment, often in tandem with hospice aides. But, patients and families experienced disruption in both usual and hospice aide services due to patient or aide illness, COVID isolation policies, and the exacerbation of ongoing workforce shortages. Aides experienced emotional strain and escalation of care responsibilities including the provision of more clinically-focused end-of-life tasks (e.g., wound care, oxygen administration) usually performed by hospice nurses. When the hospice team was unable to provide home visits, the aide became the primary source of patient information. Hospice staff supported aides in care tasks and provided emotional and spiritual care, often virtually, to aides and patients’ families. Findings highlight the critical role of aides within the care team and its intensification in a crisis, suggesting opportunities to better integrate aides into hospice care teams while providing formal training and support to help aides manage end of life care tasks and emotional demands.

